# Pirfenidone inhibits myofibroblast differentiation and lung fibrosis development during insufficient mitophagy

**DOI:** 10.1186/s12931-017-0600-3

**Published:** 2017-06-02

**Authors:** Yusuke Kurita, Jun Araya, Shunsuke Minagawa, Hiromichi Hara, Akihiro Ichikawa, Nayuta Saito, Tsukasa Kadota, Kazuya Tsubouchi, Nahoko Sato, Masahiro Yoshida, Kenji Kobayashi, Saburo Ito, Yu Fujita, Hirofumi Utsumi, Haruhiko Yanagisawa, Mitsuo Hashimoto, Hiroshi Wakui, Yutaka Yoshii, Takeo Ishikawa, Takanori Numata, Yumi Kaneko, Hisatoshi Asano, Makoto Yamashita, Makoto Odaka, Toshiaki Morikawa, Katsutoshi Nakayama, Kazuyoshi Kuwano

**Affiliations:** 1 Division of Respiratory Diseases, Department of Internal Medicine, Jikei University School of Medicine, 3-25-8 Nishi-shimbashi, Minato-ku, Tokyo, 105-8461 Japan; 2 Research Institute for Diseases of the Chest, Graduate School of Medical Sciences, Kyushu University, Fukuoka, Japan; 3 Department of Respiratory Medicine, Faculty of Life Science, Kumamoto University, Kumamoto, Japan; 4 Division of Chest Diseases; Department of Surgery, Jikei University School of Medicine, Tokyo, Japan

**Keywords:** Autophagy, IPF, Myofibroblast, Mitophagy, Pirfenidone

## Abstract

**Background:**

Pirfenidone (PFD) is an anti-fibrotic agent used to treat idiopathic pulmonary fibrosis (IPF), but its precise mechanism of action remains elusive. Accumulation of profibrotic myofibroblasts is a crucial process for fibrotic remodeling in IPF. Recent findings show participation of autophagy/mitophagy, part of the lysosomal degradation machinery, in IPF pathogenesis. Mitophagy has been implicated in myofibroblast differentiation through regulating mitochondrial reactive oxygen species (ROS)-mediated platelet-derived growth factor receptor (PDGFR) activation. In this study, the effect of PFD on autophagy/mitophagy activation in lung fibroblasts (LF) was evaluated, specifically the anti-fibrotic property of PFD for modulation of myofibroblast differentiation during insufficient mitophagy.

**Methods:**

Transforming growth factor-β (TGF-β)-induced or ATG5, ATG7, and PARK2 knockdown-mediated myofibroblast differentiation in LF were used for in vitro models. The anti-fibrotic role of PFD was examined in a bleomycin (BLM)-induced lung fibrosis model using PARK2 knockout (KO) mice.

**Results:**

We found that PFD induced autophagy/mitophagy activation via enhanced PARK2 expression, which was partly involved in the inhibition of myofibroblast differentiation in the presence of TGF-β. PFD inhibited the myofibroblast differentiation induced by PARK2 knockdown by reducing mitochondrial ROS and PDGFR-PI3K-Akt activation. BLM-treated PARK2 KO mice demonstrated augmentation of lung fibrosis and oxidative modifications compared to those of BLM-treated wild type mice, which were efficiently attenuated by PFD.

**Conclusions:**

These results suggest that PFD induces PARK2-mediated mitophagy and also inhibits lung fibrosis development in the setting of insufficient mitophagy, which may at least partly explain the anti-fibrotic mechanisms of PFD for IPF treatment.

## Background

Idiopathic pulmonary fibrosis (IPF) is a progressive fibrosing interstitial pneumonia of unknown cause with poor prognosis [1–3]. Due to the relative paucity of inflammatory cell infiltration as well as the failure of anti-inflammatory and immunosuppressive treatment modalities, the aberrant wound healing processeses represented by the formation of fibroblastic foci (FF) are recognized to be responsible for fibrotic remodeling during IPF pathogenesis [4]. Although the exact biological mechanisms for FF formation remain to be clearly determined, FF are known to be comprised of myofibroblasts with increased extracellular matrix production and a contractile phenotype. Hence, the mechanisms of myofibroblast differentiation have been recognized to be crucial targets for IPF treatment. Indeed, recently available treatments of nintedanib and pirfenidone (PFD) are considered to be effective mainly through anti-fibrotic mechanisms, including inhibition of myofibrobast differentiation and proliferation [5, 6].

PFD is a small molecule approved for the treatment of IPF worldwide and demonstrates significant reduction in the decline of forced vital capacity for IPF patients [6]. PFD is known to exert both anti-inflammatory and anti-fibrotic properties via suppressing inflammatory and pro-fibrotic cytokine expression during bleomycin-induced lung fibrosis development [7]. PFD regulates inflammatory cytokine expression through modulating cell signaling, including nuclear factor kappa B(NF-kB) and P38 mitogen-activated protein kinase (MAPK) pathways [8, 9]. Anti-fibrotic mechanisms for PFD have been postulated to be mainly attributed to attenuating protein levels and signaling pathways of pro-fibrotic growth factors, including transforming growth factor (TGF)-β, platelet-derived growth factor (PDGF), and basic-fibroblast growth factor (bFGF) [7, 10, 11]. Furthermore, PFD has been shown to have anti-oxidative properties [12–14]. Despite the wide array of pharmacological inhibition of fibrotic processes that have been reported for PFD, the exact molecular mechanisms for attenuating lung fibrosis progression remain elusive [15].

Autophagy is cellular machinery for delivering cytoplasmic components for lysosomal degradation, which occurs continuously at basal levels during homeostatic turnover. In addition, proper turnover of damaged organelles by selective autophagy is considered to be cytoprotective during the integrated stress response [16, 17]. Involvement of insufficient autophagy in IPF pathogenesis through enhancing myofibroblast differentiation has been demonstrated [18, 19]. We have recently reported that insufficient autophagy/mitophagy-mediated enhanced mitochondrial reactive oxygen species (ROS) is responsible for myofibroblast differentiation through regulation of the platelet-derived growth factor receptor (PDGFR) signaling pathway [20]. Hence, it is likely that autophagy/mitophagy modulation represents a promising modality of IPF treatment. In terms of association between autophagy and IPF treatment, nintedanib has been shown to activate ATG7 independent non-canonical autophagy, which was not involved in the anti-fibrotic mechanisms of nintedanib [21]. In the present paper, we have attempted to elucidate the participation of PFD in autophagy/mitophagy regulation and have also examined the anti-fibrotic properties of PFD during insufficient autophagy/mitophagy.

## Methods

### Cell culture, antibodies, and reagents

Human lung tissues were obtained from pneumonectomy and lobectomy specimens for primary lung cancer. Informed consent was obtained from all surgical participants as part of an approved ongoing research protocol by the ethical committee of Jikei University School of Medicine{#20-153(5443)}. Lung fibroblasts were isolated and characterized as previously described [22]. Lung fibroblasts outgrown from lung fragments were cultured in fibroblast growth media (Dulbecco's Modified Eagle's Medium (DMEM): with 10% FCS and penicillin-streptomycin). Lung fibroblasts were serially passaged and used for experiments until passage 6. LF demonstrated >95% positive staining with anti-vimentin antibodies, and <5% positive staining with anti-cytokeratin antibody (Data not shown). Antibodies used were rabbit anti-PARK2 (Cell signaling Technology, # 2132), rabbit anti-PINK1 (Cell signaling Technology, # 6946), rabbit anti-phospho-PDGF receptor α (Cell signaling Technology, # 2992), rabbit anti-PDGFRα (Santa Cruz Biotechnology, #338), mouse anti-phospho-PDGF receptor β (Cell signaling Technology, #3166), rabbit anti-PDGF receptor β (Cell signaling Technology, #3169), rabbit anti-phospho-PI3K (Cell signaling Technology, #4228), rabbit anti-PI3K (Cell signaling Technology, #4257), rabbit anti-phospho-AKT (Cell signaling Technology, #4060), rabbit anti-AKT (Cell signaling Technology, #4691), rabbit anti-phospho- p70 S6Kinase(Cell signaling Technology, #9205), rabbit anti- p70 S6Kinase (Cell signaling Technology, #9202), rabbit anti-phospho-4EBP-1 (Cell signaling Technology, #9451), rabbit anti-4EBP-1 (Cell signaling Technology, #945), rabbit anti-microtubule-associated protein 1A/1B-light chain 3 (LC3) (Novus, #600-1384), rabbit anti-ATG5 (Cell signaling Technology, #2630), rabbit anti-ATG7 (Cell signaling Technology, #2631), rabbit anti-p62 SQSTM1 (MBL, #PM045), mouse anti-α smooth muscle actin (Sigma-Aldrich, #A2547), goat anti-type I collagen (Southern Biotech, #1310-0), and mouse anti-β-actin (Sigma-Aldrich, #A5441). Bafilomycin A1 (Baf A1) (Sigma-Aldrich, #B1793), Hoechst 33258 (Sigma-Aldrich, #B2883), MitoSOX Red (Molecular probes Life technologies, #M36008), N-acetylcysteine (NAC) (Wako, # 017-05131), Mito-TEMPO (Enzo Life Sciences, #ALX-430-150), pepstatin A (Peptide Institute, #4397), E64d (Peptide Institute, #4321-v), bleomycin (Nippon Kayaku Co., Tokyo, Japan) and CM-H2DCFDA (Life Technologies, #C6827) were purchased. Pirfenidone (PFD) was provided by Shionogi & Co., Ltd. (Osaka, Japan).

### Plasmids, siRNA, and transfection

The LC3 cDNA was the kind gift of Dr. Mizushima (Tokyo University, Tokyo, Japan) and Dr. Yoshimori (Osaka University, Osaka, Japan), and was cloned into the pEGFP-C1 vector[23]. Small interfering RNA (siRNA) targeting ATG5 (Applied Biosystems Life Technologies, #s18159, s18160), ATG7 (Applied Biosystems Life Technologies, #s20650, s20651), PARK2 (Applied Biosystems Life Technologies, #s10043, s10044), and negative control siRNAs (Applied Biosystems Life Technologies, #AM4635, AM4641) were purchased from Life Technologies. Specific knock-downs of ATG5, ATG7, and PARK2 were validated using two different siRNA, respectively. Transfections of LF were performed using the Neon® Transfection System (Invitrogen Life Technologies, #MPK5000), using matched optimized transfection kits (Invitrogen Life Technologies, #MPK10096).

### RNA isolation, polymerase chain reaction

RNA isolation, reverse transcription and Real-Time PCR were performed using the SYBR green method as previously described [22]. The primers used were PARK2 sense primer, 5’-AAATGCCCAGACAAGATGCC-3’; PARK2 antisense primer, 5’-GGCCTCTCACGACTGAGTT-3’; ACTB sense primer 5’-CATGTACGTTGCTATCCAGGC-3’ ACTB antisense primer 5’-CTCCTTAATGTCACGCACGAT-3’. These primer sets yielded PCR products of 135 bp and 250 bp for PARK2 and ACTB respectively. PCRs of PARK2 were validated using two different primers. Primer sequences were from Primer Bank (http://pga.mgh.harvard.edu/primerbank.)

### Measurement of ROS production

LF, at a density of 1 X10^4^ per well, were seeded in a 96-well microplate (Thermo Fisher Scientific, # 237105). CM-H2DCFDA was used to measure total cellular ROS according to the manufacturer's instructions. After incubation with CM-H2DCFDA (10 μM) for 30 min at 37 °C, fluorescence of DCF was measured at an excitation wavelength of 485 nm and an emission wavelength of 535 nm by a fluorescence microplate reader (Infinite F 200) (Tecan Japan, Kanagawa, Japan). Mitochondrial ROS production was analyzed by MitoSOX Red staining according to the manufacturer’s instructions, which was evaluated by fluorescence microscopy (Olympus, Tokyo, Japan and Keyence, BZ-X700).

### Western blotting

LF grown on 6-well culture plates were lysed in RIPA buffer (Thermo Fisher Scientific, catalog # 89900) with protease inhibitor cocktail (Roche Diagnostics, # 11697498001) and 1 mM sodium orthovanadate, or lysed with Laemmli sample buffer. Western blotting was performed as previously described [20]. For each experiment, equal amounts of total protein were resolved by 7.5‐15% SDS/PAGE. After SDS/PAGE, proteins were transferred to polyvinylidene difluoride (PVDF) membrane (Millipore, # ISEQ00010), and incubation with specific primary antibody was performed for 2 h at 37 °C, or 24 h at 4 °C. After washing several times with PBST, the membrane was incubated with Anti-rabbit IgG, HRP-linked secondary antibody (Cell Signaling Technology, # 7074), Anti-mouse IgG, HRP-linked secondary antibody, # 7076) or Anti-goat IgG (H + I), HRP-linked secondary antibody (BETHYL, #A50-100P) followed by chemiluminescence detection (Thermo scientific, # 34080, and BIO-RAD, # 1705061) with the ChemiDocTM Touch Imaging System (BIO-RAD, California, USA).

### Mitochondria isolation

Mitochondria and cytosolic fractions were isolated from LF by a commercially available kit (Thermo Fisher Scientific, #89874) according to the manufacturer's instructions.

### Immunofluorescence staining

LF were transfected with pEGFP-LC3 with a concomitant non-silencing control siRNA, ATG5 siRNA, ATG7 siRNA, or PARK2 siRNA and PFD treatment was started 48 h post-transfection. Baf A1 (20 nM) treatment was started 6 h before fixation to clearly demonstrate the autophagosome formation of GFP-LC3 “dots”, which result from BafA1 prevention of lysosomal degradation. After 24 h treatment with PFD, lung fibroblasts were fixed with 4% paraformaldehyde for 15 minutes followed by permeabilization with 0.03% Triton X (Wako, # 160-24751) for 60 min. After blocking with 1% BSA (Sigma-Aldrich, #A2153) for 60 min, the primary and secondary antibodies were applied according to the manufacturer’s instructions. Confocal laser scanning microscopic analysis (ZEISS, LSM-880) of mitochondria was performed by mouse anti-TOM20 (Santa Cruz, #17764) staining.

### Mouse models

C57BL/6J (CLEA Japan INC, Tokyo, Japan) and B6.129S4-Park2tm1Shn/J (Jackson Laboratories, Bar Harbor, ME) mice were purchased, and were maintained in the animal facility at the Jikei University School of Medicine (#25031). All experimental procedures are approved by the Jikei University School of Medicine Animal Care Committee. A dose of 2.5 U/kg bleomycin (Nippon Kayaku Co., Tokyo, Japan) was intratracheally administered in 50 μL saline. Intraesophageal administration of PFD (9.25 mg/body/day) or 0.5% DMSO were given on days 1 to 20. The lungs were removed at day 21 and were used for histological examination and Sircol soluble collagen assay (biocolor, #S1000). For histological examination, the lungs were fixed overnight in 10% buffered formalin, embedded in paraffin, and sections stained with Hematoxylin & Eosin (H-E) and Masson’s trichrome according to conventional methods for histopathological evaluation. Immunohistochemistry was performed as previously described [20]. Quantitative measure of Masson’s trichrome staining was performed by using Image J, an open source image processing program. For quantitatively monitoring collagen production in mouse lung, Sircol soluble collagen assay of left lungs was performed according to the manufacturer's instructions.

### Statistics

Data are shown as the average (±SEM) taken from at least three independent experiments. Student’s *t*-test was used for comparison of two data sets, analysis of variance for multiple data sets. Tukey’s or Dunn’s test were used for parametric and nonparametric data, respectively, to find where the difference lay. Significance was defined as *p* < 0.05. Statistical software used was Prism v.5 (GraphPad Software, Inc., San Diego, CA).

## Results

### PFD activates autophagy in LF

**Fig. 1:**
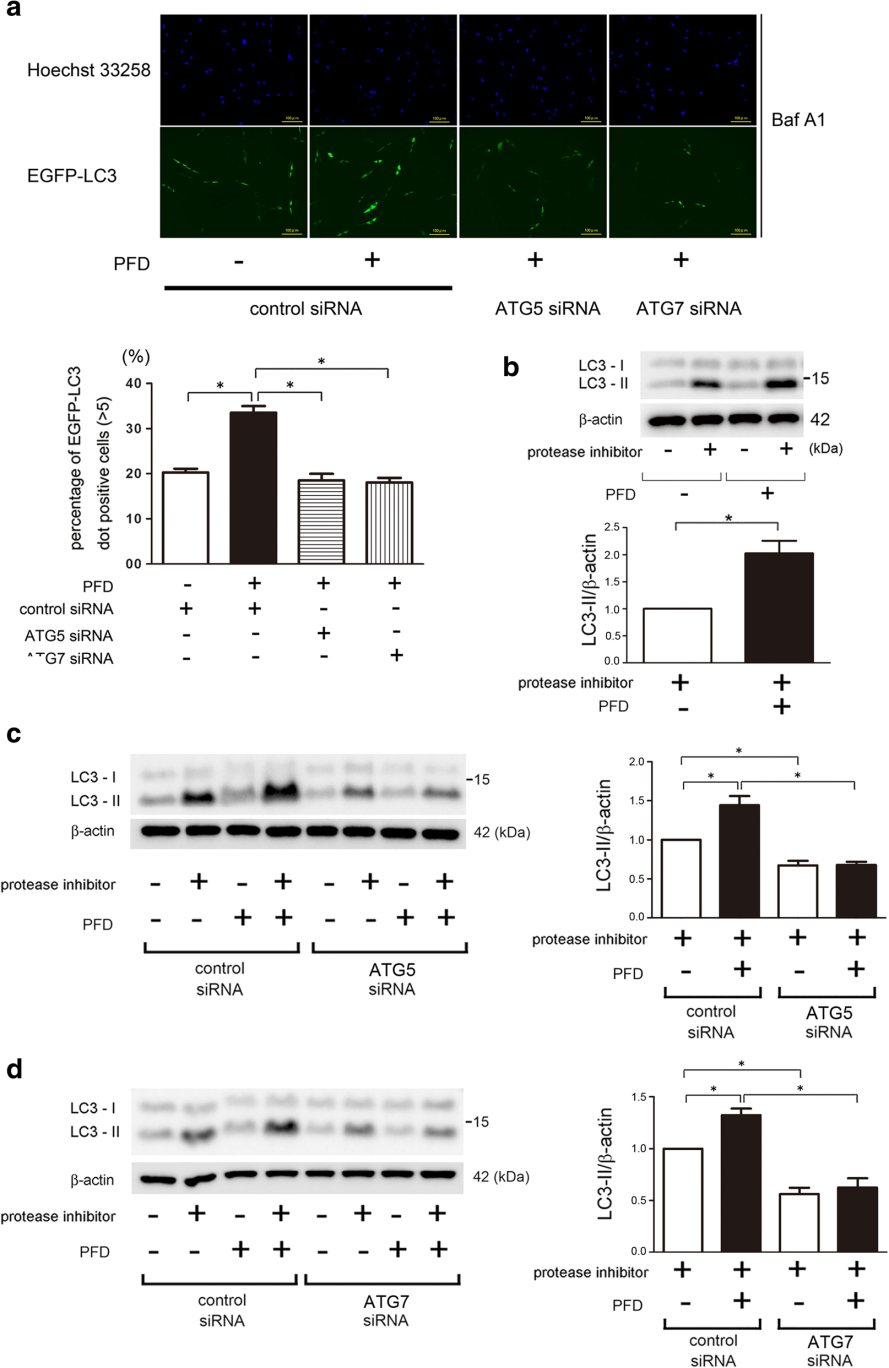
Autophagy activation by PFD in LF. **a** Fluorescence microscopic detection of pEGFP-LC3 dot formation in LF: LF were transfected with pEGFP-LC3 and control siRNA, ATG5 siRNA, or ATG7 siRNA. Photomicrographs are taken at the same magnification. (Original magnification, 200X). The *lower panel* is the percentage of positive cells with more than *five dot formations* (±SEM) and data was collected from four independent experiments. *p < 0.05. **b** Western blotting (WB) using anti-LC3 and anti-β-actin of cell lysates from control (lane 1, 2), PFD (500 μg/ml) (lane 3, 4) treated LF. LF were treated with PFD for 24 h and protease inhibitor (E64d 10 μg/ml, pepstatin A 10 μg/ml) treatment was started 6 h before collecting cell lysates. In the *lower panel* is the average (±SEM) taken from six independent experiments shown as relative expression. *p < 0.05. **c** WB using anti-LC3 and anti-β-actin of cell lysates from control siRNA (lane 1 ~ 4), ATG5 siRNA (lane 5 ~ 8) transfected LF. PFD (500 μg/ml) treatment was started 48 h post transfection and protein samples were collected after 24 h treatment. Protease inhibitor (E64d 10 μg/ml, pepstatin A 10 μg/ml) treatment was started 6 h before collecting cell lysates. In the *right panel* is the average (±SEM) taken from five independent experiments shown as relative expression. *p < 0.05. **d** WB using anti-LC3 and anti-β-actin of cell lysates from control siRNA (lane 1 ~ 4), ATG7 siRNA (lane 5 ~ 8) transfected LF. PFD(500 μg/ml) treatment was started 48 h post transfection and protein samples were collected after 24 h treatment. Protease inhibitor (E64d 10 μg/ml, pepstatin A 10 μg/ml) treatment was started 6 h before collecting cell lysates. In the *right panel* is the average (±SEM) taken from five independent experiments shown as relative expression. *p < 0.05

Firstly, autophagy in response to PFD was examined in lung fibroblasts (LF) isolated from normal lungs. Previous papers showing inhibition of TGF-β-induced myofibroblast differentiation used 500 μg/ml of PFD [24, 25], which is 50 fold higher than maximum drug concentrations observed in the blood. Our preliminary experiments also demonstrated that 500 μg/ml of PFD was needed to efficiently inhibit TGF-β-induced myofibroblast differentiation in LF (data not shown), hence 500 μg/ml of PFD was selected for in vitro experiments. Autophagy activation was evaluated by using LF expressing EGFP-LC3 and increase in EGFP-LC3 dots reflecting autophagosome formation was observed following PFD treatment (Fig. 1a). PFD-mediated autophagy was further confirmed by detecting the conversion of LC3 from LC3-I (free form) to LC3-II (phosphatidylethanolamine-conjugated form), which is a crucial step during autophagosome formation (Fig. 1b). Protease inhibitor (E64d and pepstatin A) treatment was started 6 h before sample collection to precisely evaluate LC3-II accumulation by preventing lysosomal degradation. A recent paper demonstrated that nintedanib activates ATG7-independent but Beclin1-dependent non-canonical autophagy [21]. To clarify whether PFD-induced autophagy is canonical or not, siRNA-mediated knockdown of ATG5 and ATG7 was performed. Both ATG5 and ATG7 knockdown apparently suppressed PFD-induced autophagy by means of EGFP-LC3 dot formation and conversion of LC3 (Fig. 1a, c, d), indicating canonical autophagy activation by PFD.

### PFD induces PARK2-dependent mitophagy in LF

**Fig. 2:**
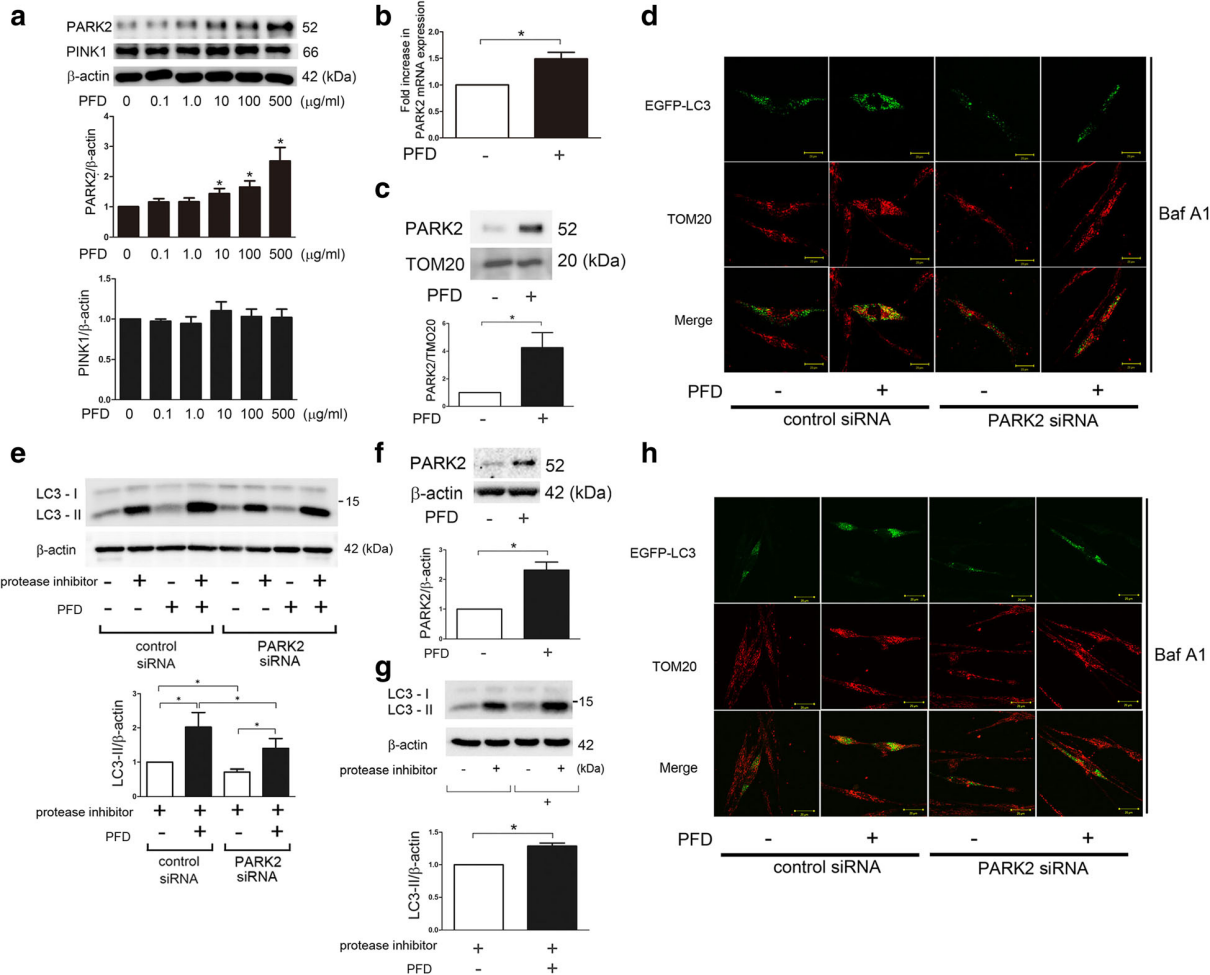
PARK2-mediated mitophagy activation by PFD in LF. **a** WB using anti-PARK2, anti-PINK1, and anti-β-actin of cell lysates from control (lane 1) and indicated concentrations of PFD (lane2 ~ 6) treated LF. Protein samples were collected after 24 h treatment with PFD. In the lower panels are the average (±SEM) taken from six independent experiments shown as relative expression. *p < 0.05. **b** LF were treated with PFD (500 μg/ml) and mRNA samples were collected after 24 h treatment with PFD (*n* = 4). Real-Time-PCR was performed using primers to PARK2 or β-actin, as a control. PARK2 mRNA expression was normalized to β-actin. Shown is the fold increase (±SEM) relative to control treated cells. *p < 0.05. **c** WB using anti-PARK2 and anti-TOM20 of cell lysates from PFD (500 μg/ml)-treated LF. Protein samples for mitochondrial fractions were collected after 24 h treatment. The *lower panel* is the average (±SEM) taken from three independent experiments shown as relative expression. *p < 0.05. **d** Colocalization analysis of confocal laser scanning microscopic images of TOM20 staining and EGFP-LC3. LF were transfected with pEGFP-LC3 with a concomitant non-silencing control siRNA or PARK2 siRNA. PFD (500 μg/ml) treatment was started 48 h post-transfection. Baf A1 (20 nM) treatment was started 6 h before fixation and LF were fixed after 24 h treatment with PFD. The images are high magnification (400X). (Bar = 20 μm). **e** WB using anti-LC3 and anti-β-actin of cell lysates from control siRNA (lane 1 ~ 4), PARK2 siRNA (lane 5 ~ 8) transfected LF. PFD (500 μg/ml) treatment was started 48 h post transfection and protein samples were collected after 24 h treatment. Protease inhibitor (E64d 10 μg/ml, pepstatin A 10 μg/ml) treatment was started 6 h before collecting cell lysates. In the *right panel* is the average (±SEM) taken from four independent experiments shown as relative expression. *p < 0.05. **f** WB using anti-PARK2 and anti-β-actin of cell lysates from PFD (500 μg/ml)-treated LF isolated from IPF lungs. Protein samples were collected after 24 h treatment. The lower panel is the average (±SEM) taken from six independent experiments shown as relative expression. *p < 0.05. **g** WB using anti-LC3 and anti-β-actin of cell lysates from control (lane 1, 2), PFD (500 μg/ml) (lane 3, 4) treated LF isolated from IPF lungs. LF were treated with PFD for 24 h and protease inhibitor (E64d 10 μg/ml, pepstatin A 10 μg/ml) treatment was started 6 h before collecting cell lysates. In the *lower panel* is the average (±SEM) taken from six independent experiments shown as relative expression. *p < 0.05. (H) Colocalization analysis of confocal laser scanning microscopic images of TOM20 staining and EGFP-LC3. LF isolated from IPF lungs were transfected with pEGFP-LC3 with a concomitant non-silencing control siRNA or PARK2 siRNA. PFD (500 μg/ml) treatment was started 48 h post-transfection. Baf A1 (20 nM) treatment was started 6 h before fixation and LF were fixed after 24 h treatment with PFD. The images are high magnification (400X). (Bar = 20 μm)

The phosphatase and tensin homolog (PTEN)-induced putative protein kinase 1 (PINK1) and PARK2 pathway has been largely implicated in the mitochondria selective autophagy known as mitophagy. PINK1 stabilizes on the mitochondrial outer membrane during stress-induced mitochondrial membrane depolarization, resulting in recruitment of PARK2 to the mitochondria. Ubiquitination of mitochondrial proteins by PARK2, an E3 ubiquitin ligase, is required for conducting mitophagy. Our recent paper elucidated that PARK2-mediated mitophagy is responsible for regulation of myofibroblast differentiation in LF [20]. Hence, the involvement of PARK2-mediated mitophagy was examined during PFD treatment. Intriguingly PFD apparently induced PARK2 protein expression in a dose dependent manner and significant increase was observed at the concentration of 10 μg/ml (Fig. 2a). In contrast, no apparent change was demonstrated in PINK1 protein levels (Fig. 2a). RT-PCR elucidated that PFD-mediated PARK2 protein expression was reflected by mRNA levels (Fig. 2b). Accumulation of PARK2 in the mitochondrial fraction was also observed, suggesting PFD-mediated increase in PARK2 may be associated with enhanced mitophagy (Fig. 2c). Confocal microscopy was performed in LF expressing EGFP-LC3, and colocalization of TOM20-stained mitochondria and EGFP-LC3 dots was used to characterize mitophagy. Colocalization was detected in the presence of bafilomycin A (Baf A1), an inhibitor of autolysosomal degradation, and PFD treatment clearly enhanced colocalization of TOM20 and EGFP-LC3, reflecting PFD-induced mitophagy (Fig. 2d). SiRNA-mediated PARK2 knockdown experiments showed reduced colocalization of TOM20 and EGFP-LC3, indicating that PFD-mediated mitophagy can be attributed to the enhanced PARK2 protein levels (Fig. 2d). Furthermore, PARK2 plays an important role not only in mitophagy but also in conducting autophagy itself through interacting with Beclin1 [26], thus autophagy activation by PFD was evaluated in the setting of PARK2 knockdown. PFD-induced LC3 conversion was significantly reduced by PARK2 knockdown, indicating that autophagy activation by PFD was at least partly conferred by enhanced PARK2 protein levels (Fig. 2e).

PFD-induced PARK2 protein expression and autophagy/mitophagy were further confirmed in LF isolated from IPF lungs, suggesting potential therapeutic implication of PFD-mediated autophagy/mitophagy in IPF pathogenesis (Fig. 2f, g, h).

### PFD attenuates myofibroblast differentiation induced by TGF-β treatment and by autophagy/mitophagy inhibition in LF

**Fig. 3:**
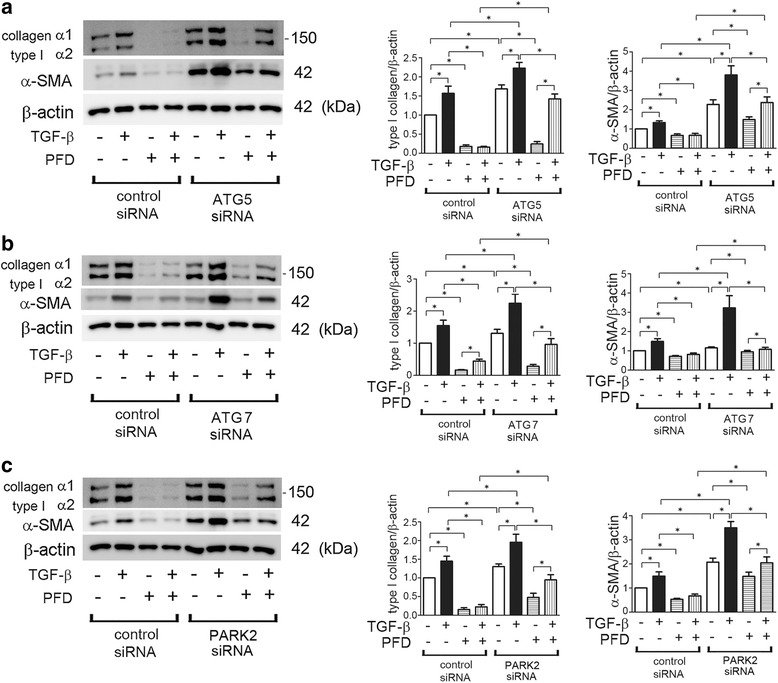
PFD attenuates myofibroblast differentiation during insufficient autopagy/mitophagy in LF. **a** WB using anti-type I collagen, anti-α-SMA, and anti-β-actin of cell lysates from control siRNA (lane 1 ~ 4), ATG5 siRNA (lane 5 ~ 8) transfected LF. TGF-β (2 ng/ml) and PFD (500 μg/ml) treatment was started 48 h post transfection and protein samples were collected after 24 h treatment. In the *right panels* are the average (±SEM) taken from five independent experiments shown as relative expression. *p < 0.05. **b** WB using anti-type I collagen, anti-α-SMA, and anti-β-actin of cell lysates from control siRNA (lane 1 ~ 4), ATG7 siRNA (lane 5 ~ 8) transfected LF. TGF-β (2 ng/ml) and PFD (500 μg/ml) treatment was started 48 h post transfection and protein samples were collected after 48 h treatment. In the *right panels* are the average (±SEM) taken from five independent experiments shown as relative expression. *p < 0.05. **c** WB using anti-type I collagen, anti-α-SMA, and anti-β-actin of cell lysates from control siRNA (lane 1 ~ 4), PARK2 siRNA (lane 5 ~ 8) transfected LF. TGF-β (2 ng/ml) and PFD (500 μg/ml) treatment was started 48 h post transfection and protein samples were collected after 24 h treatment. In the *right panels* are the average (±SEM) taken from five independent experiments shown as relative expression. *p < 0.05

To clarify whether PFD-induced autophagy/mitophagy is responsible for inhibition of myofibroblast differentiation in LF, ATG5, ATG7, and PARK2 knockdown experiments were performed, respectively. PFD markedly suppressed type I collagen and αSMA expression, (markers of myofibroblast differentiation), regardless of TGF-β treatment in control siRNA transfected LF (Fig. 3a, b, c). In line with our recent report, ATGs and PARK2 knockdown of autophagy/mitophagy inhibition induced myofibroblast differentiation, which was further enhanced by TGF-β treatment [19, 20]. Intriguingly, there are significant differences in the extent of myofibroblast inhibition by PFD between control and ATGs/PARK2 knockdown in the presence of TGF-β (lane 4 and 8), suggesting the potential participation of autophagy/mitophagy in PFD-mediated myofibroblast inhibition. However, PFD inhibited TGF-β-induced myofibroblast differentiation in ATGs and PARK2 knockdown comparable to levels shown in ATGs and PARK2 knockdown without TGF-β, suggesting that PFD-induced autophagy/mitophagy is regulating myofibroblast differentiation beyond that induced by TGF-β.

### PFD suppresses ROS production and activation of PI3K/Akt signaling pathway during insufficient mitophagy in LF

**Fig. 4:**
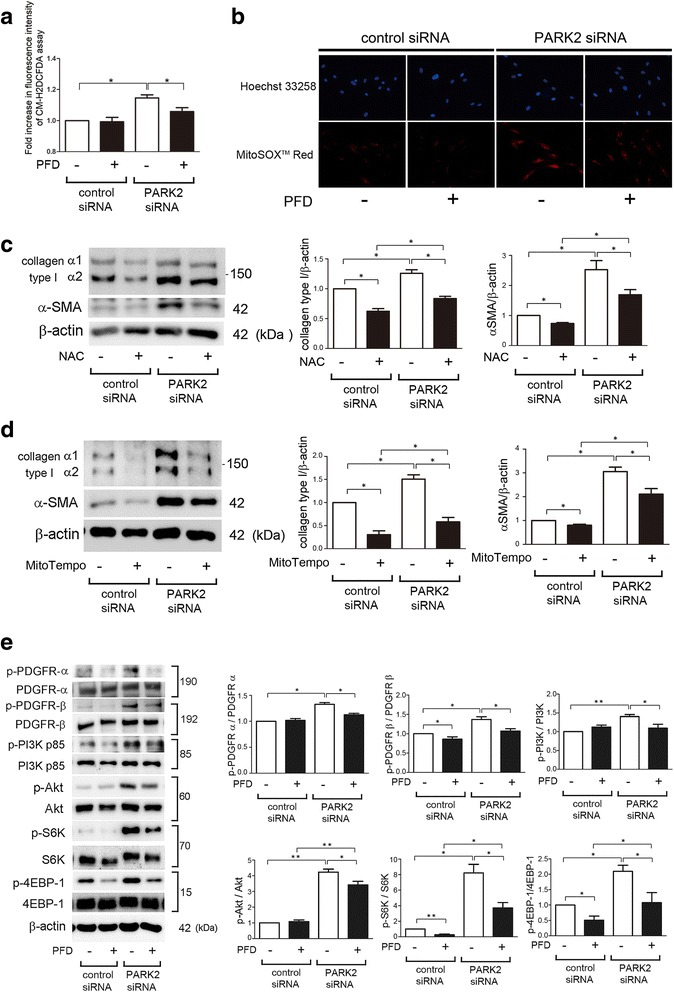
PFD attenuates myofibroblast differentiation during insufficient mitophagy via inhibiting PDGFR/PI3K/AKT signaling pathway in LF. **a** Fluorescence intensity of CM-H2DCFDA staining for intracellular ROS production. PFD (500 μg/ml) treatment was started 48 h post-siRNA transfection and incubation with CM-H2DCFDA (10 μM) was started after 24 h treatment in LF. The fluorescence level in the control siRNA transfected cells without PFD was designated as 1.0. The panel is the average (±SEM) taken from five independent experiments shown as relative expression.* *p* < 0.05. **b** Photographs of Hoechst 33258 and MitoSOX Red fluorescence staining in LF. LF were transfected with control siRNA and PARK2 siRNA. PFD (500 μg/ml) treatment was started 48 h post-siRNA transfection and staining was performed 48 h transfection. (Original magnification, 200X) (**c**) WB using anti-type I collagen, anti-α-SMA, and anti-β-actin of cell lysates from control siRNA (lane 1, 2), PARK2 siRNA (lane 3, 4) transfected LF. NAC (1 mM) treatment was started 48 h post transfection and protein samples were collected after 24 h treatment. In the *right panels* are the average (±SEM) taken from six independent experiments shown as relative expression. *p < 0.05. **d** WB using anti-type I collagen, anti-α-SMA, and anti-β-actin of cell lysates from control siRNA (lane 1, 2), PARK2 siRNA (lane 3, 4) transfected LF. MitoTempo (100 μM) treatment was started 48 h post transfection and protein samples were collected after 24 h treatment. In the right panels are the average (±SEM) taken from six independent experiments shown as relative expression. *p < 0.05. **e** WB using anti-phospho-PDGFR-α (p-PDGFR-α), anti-PDGFR-α, anti-phospho-PDGFR-β (p-PDGFR-β), anti-PDGFR-β, anti-phospho-PI3K p85 (p-PI3K p85), anti-PI3K p85, anti- phospho-AKT (p-AKT), anti-AKT, anti-phopho-S6K (p-S6K), anti-S6K, anti-phospho-4EBP-1 (p-4EBP-1), anti-4EBP-1, and anti-β-actin of cell lysates from control siRNA (lane 1, 2) and PARK2 siRNA(lane 3, 4) transfected LF. PDF (500 μg/ml) treatment was started 48 h post transfection and protein samples were collected after 24 h treatment. In the *lower right panels* are the average (±SEM) taken from five independent experiments shown as relative expressions. *p < 0.05

To elucidate the anti-fibrotic mechanisms of PFD, next we focused on the anti-oxidative properties of PFD [13, 14]. Recent papers, including our findings, propose a role for the accumulation of mitochondrial ROS in IPF pathogenesis due to impaired mitophagy [20, 27]. We have recently reported that increased mitochondrial ROS is responsible for activating the PDGFR–PI3K-AKT pathway, resulting in myofibroblast differentiation in the setting of insufficient mitophagy [20]. Accordingly, the causal link between the anti-oxidative property of PFD and inhibition of myofibroblast differentiation was examined by using PARK2 knockdown LF. Mitophagy inhibition by PARK2 knockdown-mediated ROS production was clearly shown by means of the CM-H2DCFDA assay for detecting intracellular ROS and Mitosox Red staining for mitochondrial superoxide, respectively (Fig. 4a, b). PFD clearly suppressed intracellular ROS and mitochondrial superoxide production during PARK2 knockdown (Fig. 4a, b). Both NAC (an antioxidant for intracellular ROS) and Mito-TEMPO (a specific antioxidant for mitochondrial ROS) efficiently inhibited myofibroblast differentiation of type I collagen and αSMA expression caused by PARK2 knockdown (Fig. 4c, d), suggesting that the anti-oxidative properties of PFD result mainly from regulation of mitophagy.

Next we examined the involvement of PFD in the regulation of PDGFR-PI3K-AKT-mTOR signaling during myofibroblast differentiation in the setting of PARK2 knockdown. Consistent with our recent report, PARK2 knockdown activated PDGFR-PI3K-AKT-mTOR signaling [20], which was efficiently suppressed by treatment with PFD (Fig. 4e). Taken with the observations above, PFD attenuates mitochondrial ROS-mediated activation of PDGFR-PI3K-AKT-mTOR and thus inhibits myofibroblast differentiation during insufficient mitophagy.

### PFD attenuates enhanced lung fibrosis development by bleomycin (BLM) treatment in PARK2 knockout mouse

**Fig. 5:**
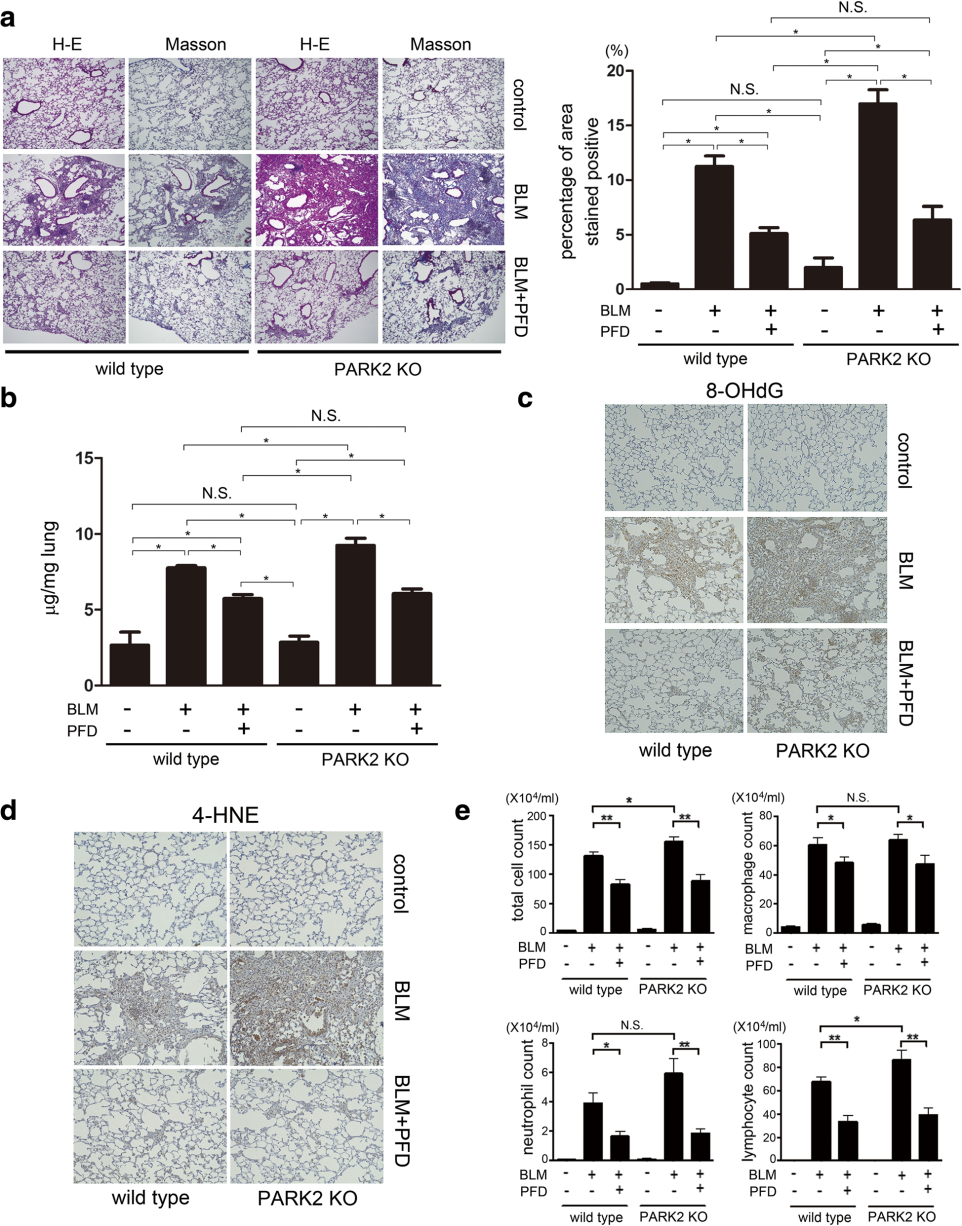
PFD attenuates bleomycin (BLM)-induced lung fibrosis development in PARK2 KO mice. **a** Photomicrographs of Hematoxylin & Eosin (H-E) and Masson trichrome staining of mouse lungs at day 21. Original magnification × 200. The *right panel* shows the average (±SEM) percentage of positively stained areas in Masson trichrome staining quantified using Image J. Treatment groups were composed of vehicle treated wild type (*n* = 10), BLM-treated wild type (*n* = 10), BLM-treated wild type with subsequent PFD (*n* = 10), BLM-treated PARK2 KO (*n* = 6), and BLM-treated PARK2 KO with subsequent PFD (*n* = 6). *p < 0.05. N.S.: not statistically significant. **b** Shown in the panel is the average (±SEM) soluble collagen measurement from Sircol assay using vehicle treated wild type (*n* = 10), BLM-treated wild type (*n* = 10), BLM-treated wild type with subsequent PFD (*n* = 10), BLM-treated PARK2 KO (*n* = 6), and BLM-treated PARK2 KO with subsequent PFD (*n* = 6) at day 21. *p < 0.05. N.S.: not statistically significant. **c** Immunohistochemical staining of 8-hydroxy-2-deoxyguanosine (8-OHdG), oxidized derivative of deoxyguanosine. Original magnification × 200 (**d**) Immunohistochemical staining of 4-hydroxy-2-nonenal (4-HNE) of lipid peroxidation. Original magnification × 200 (E) Cell counts in broncho-alveolar lavage fluid (BALF). BALF collection was performed at day 21. Cells were counted using a hemocytometer. Differential cell counts in BALF were analyzed from 300 cells stained with Diff-Quick. *p < 0.05, **p < 0.001. N.S.: not statistically significant

To further confirm the anti-fibrotic role of PFD in the setting of enhanced mitochondrial ROS resulting from insufficient mitophagy, we employed a bleomycin (BLM)-induced lung fibrosis model in PARK2 knockout (KO) mice. Compared to BLM-treated wild type mice, BLM-treated PARK2 KO mice demonstrated enhanced lung fibrosis, which was evaluated by means of Masson trichrome staining and sircol collagen assay at day 21 (Fig. 5a, b). Enhanced lung fibrosis in BLM-treated PARK2 KO mouse was effectively attenuated by treatment with PFD, which was comparable to levels seen in BLM-treated, PFD-treated wild type mice (Fig. 5a, b). This observation shows PFD attenuates lung fibrosis associated with insufficient mitophagy due to PARK2 KO. Potential anti-oxidative properties of PFD during attenuation of lung fibrosis development were examined by detecting oxidative modifications in mouse lungs. BLM treatment clearly induced expression levels of 8-hydroxy-2-deoxyguanosine (8-OHdG), the oxidized derivative of deoxyguanosine and a marker for DNA damage, as well as 4-hydroxy-2-nonenal (4-HNE), a marker of lipid peroxidation (Fig. 5c, d). Both were further enhanced in BLM-treated PARK2 KO mice. PFD treatment clearly reduced those oxidative modifications (Fig. 5c, d).

**Fig. 6:**
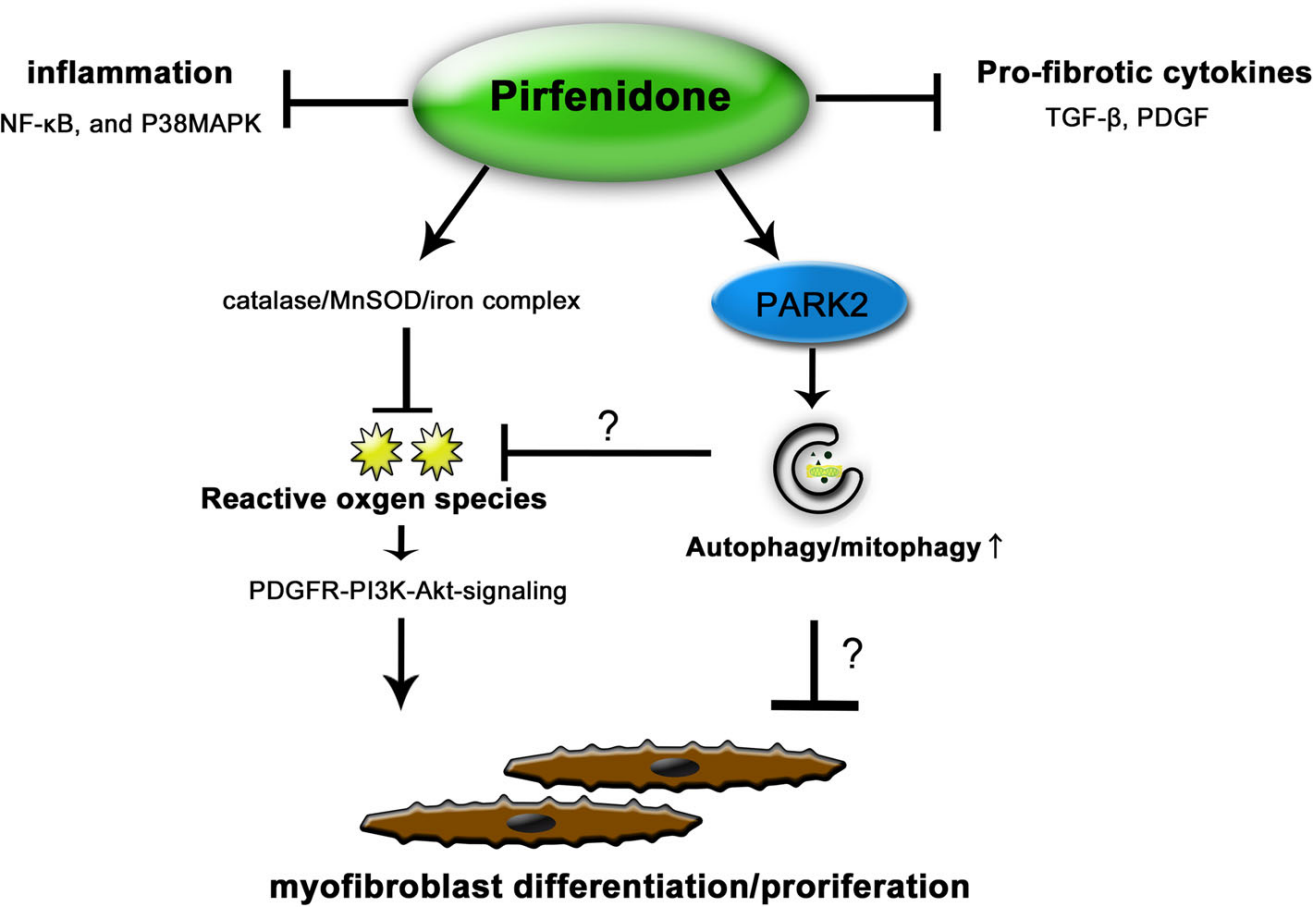
Hypothetical model of the anti-fibrotic mechanisms of PFD. PFD has previously reported anti-fibrotic mechanisms, including anti-inflammation, anti-pro-fibrotic cytokines, and anti-oxidative properties. Now PARK2-mediated autophay/mitophgy can be included in the anti-fibrotic properties of PFD. Although detailed mechanism remains elusive, PFD attenuates lung fibrosis seen during insufficient mitophagy through regulation of PDGFR-PI3K-Akt signaling by inhibiting mitochondrial ROS production

PFD has been reported to attenuate BLM-induced lung fibrosis via suppression of inflammatory cytokine expression [7]. To evaluate inflammatory cell infiltrations in lungs, we performed cell counting of bronchoalveolar lavage fluid (BALF) at day 21. BLM treatment significantly increased total cell, macrophage, neutrophil, and lymphocyte counts in wild type mice, which were further enhanced in BLM-treated PARK2 KO mice (Fig. 5e). This enhanced accumulation of inflammatory cells in BLM-treated PARK2 KO mice was reduced by treatment with PFD, which was comparable to levels seen in BLM-treated wild type mice with PFD treatment (Fig. 5e), suggesting that not only inhibition of myofibroblast differentiation but also regulation of inflammation can play a role in PFD-mediated attenuation of BLM-induced lung fibrosis.

## Discussion

In the present study, we show that PFD induces autophagy/mitophagy in part via increasing PARK2 protein levels. PFD-mediated autophagy/mitophagy regulates myofibroblast differentiation beyond that induced by TGF-β, and is at least partly responsible for the PFD-mediated inhibition of myofibroblast differentiation in LF. The anti-fibrotic properties of PFD during insufficient mitophagy of PARK2 knockdown were mainly attributed to anti-oxidative effects, resulting in attenuation of PDGFR-PI3K-AKT signaling, suggesting PARK2-mediated mitophagy is not indispensable for anti-oxidative properties of PFD. (Fig. 6). The PARK2 KO mouse model further confirmed the anti-fibrotic properties of PFD by demonstrating attenuation of oxidative modifications in the setting of insufficient mitophagy.

PFD exerts a wide array of biological effects, specifically anti-oxidative properties [7–11], including catalase expression, enhancement of manganese superoxide dismutase (MnSOD), and enhancement of scavenging superoxides [12–14]. Among those anti-oxidative mechanisms, it is very intriguing that PFD-iron complex demonstrates efficient superoxide scavenging activity at relatively lower concentrations, which is comparable to maximum drug concentrations observed in the blood [14]. We have elucidated that PFD efficiently suppressed mitochondrial ROS production in LF during insufficient mitophagy (Fig. 4). It has been reported that more than 5% of electrons transferred to mitochondria for adenosine triphosphate (ATP) production can be released as superoxide [28], indicating that mitochondria can be a main source of intrinsic superoxide, especially in the setting of enhanced mitochondrial damage such as insufficient mitophagy. Iron deposition in IPF lung has been reported in association with pulmonary hypertension [29]. Higher iron-dependent oxygen radical generation in BAL cells has been demonstrated in IPF patients [30] and interaction of ferrous iron (Fe^2+^) with H_2_O_2_ generates highly reactive hydroxyl radicals through the Fenton reaction. Accordingly, we speculate that PFD-iron complex formation can be an important molecular mechanism for lung fibrosis attenuation through reducing both free iron and mitochondrial ROS in IPF lungs where there is increased iron deposition and insufficient mitophagy [27, 30].

Autophagy has been widely implicated in IPF pathogenesis and recent reports, including our findings, have shown that insufficient mitophagy is involved in IPF pathogenesis through enhancing apoptosis and cellular senescence in epithelial cells and enhancing myofibroblast differentiation in LF [18, 19, 27]. Hence, it is likely that autophagy/mitophagy modulation can be a promising target for IPF treatment. In contrast to nintedanib, PFD induced ATG7 and ATG5-dependent canonical autophagy in LF (Fig. 1). Furthermore, PFD-induced PARK2 expression is involved in the mechanisms for autophagy and mitophagy activation by PFD (Fig. 2). Mitophagy has an essential role in keeping mitochondrial integrity through degradation of damaged mitochondria and the protective effect of PFD on mitochondorial structure has been demonstrated in proximal tubular cells [13], suggesting the potential participation of PFD-mediated mitophagy in keeping mitochondrial integrity in the setting of cellular damage. Furthermore, our knockdown experiments showed that PFD-induced autophagy/mitophagy activation is not responsible for inhibiting TGF-β-induced myofibroblast differentiation but was at least partly involved in the mechanisms for PFD-mediated myofibroblast inhibition in LF (Fig. 3). Although a direct link between PFD-mediated autophagy/mitophagy and lung fibrosis attenuation remains elusive, it is interesting to note that a significant increase in PARK2 expression levels was detected at the concentration of 10 μg/ml PDF, which is comparable to levels of maximum plasma concentration of PFD in the clinical settings. Therefore the clinical PFD dose could be sufficient to enhance PARK2 protein levels, which may exert beneficial effects via mitophagy activation and maintaining mitochondrial integrity during IPF pathogenesis.

In line with our recent findings, PARK2 KO mice demonstrated enhanced lung fibrosis in response to BLM, which was efficiently attenuated by treatment with PFD (Fig. 5). These data suggest that insufficient mitophagy is involved in the mechanisms for worsening of lung fibrosis and that PARK2-mediated mitophagy is not necessary to evoke the anti-fibrotic activities of PFD at least in BLM-induced lung fibrosis development.

However, a potential beneficial role for PFD-mediated PARK2-independent autophagy/mitophagy has not been entirely excluded. Therapeutic involvement of the anti-oxidative properties of PFD in attenuating lung fibrosis development in the setting of insufficient mitophagy was further confirmed by detecting reduced oxidative modifications in BLM-treated lungs, including 8-OHdG and 4-HNE staining, respectively (Fig. 5).

### Conclusions

Taken together, it is likely that anti-oxidative properties of PFD play an important role in attenuating lung fibrosis development in the case of insufficient mitophagy. Although autophagy/mitophagy activation by PFD was clearly demonstrated, its involvement in keeping mitochondrial integrity and attenuating lung fibrosis development during IPF pathogenesis should be precisely examined in future studies.

## Abbreviations

4-HNE: 4-hydroxy-2-nonenal
8-OHdG: 8-hydroxy-2-deoxyguanosine
ATP: Adenosine triphosphate
BALF: Bronchoalveolar lavage fluid
bFGF: basic-fibroblast growth factor
BLM: Bleomycin
CM-H2DCFDA: Chloromethyl derivative of 2′, 7′-dichlorodihydrofluorescein diacetate
DCF: 2’, 7’-Dichlorodihydrofluorescein
DMEM: Dulbecco's Modified Eagle’s Medium
ECM: Extracellular matrix
Fe^2+^: Ferrous iron
FF: Fibroblastic foci
HE staining: Hematoxylin-Eosin staining
IPF: Idiopathic pulmonary fibrosis
KO: Knockout
LF: Lung fibroblasts
MAPK: Mitogen-activated protein kinase
MnSOD: Manganese superoxide dismutase
NAC: N-acetylcysteine
NF-kB: Nuclear factor kappa B
PDGF: Platelet-derived growth factor
PDGFR: Platelet-derived growth factor receptor
PFD: Pirfenidone
PI3K: Phosphoinositide 3-kinase
PVDF: Polyvinylidene difluoride
ROS: Reactive oxygen species
SEM: Standard error of the mean
siRNA: small interfering RNA
TGF-β: Transforming growth factor-β
WB: Western blotting
α-SMA: α-smooth muscle actin
